# Headache in Pregnancy: An Approach to Emergency Department Evaluation and Management

**DOI:** 10.5811/westjem.2015.1.23688

**Published:** 2015-02-25

**Authors:** Jessica C. Schoen, Ronna L. Campbell, Annie T. Sadosty

**Affiliations:** * Alpert Medical School of Brown University, Department of Emergency Medicine, Providence, Rhode Island; † Mayo Clinic, Department of Emergency Medicine, Rochester, Minnesota

## Abstract

Headache is a common presenting complaint in the emergency department. The differential diagnosis is broad and includes benign primary causes as well as ominous secondary causes. The diagnosis and management of headache in the pregnant patient presents several challenges. There are important unique considerations regarding the differential diagnosis, imaging options, and medical management. Physiologic changes induced by pregnancy increase the risk of cerebral venous thrombosis, dissection, and pituitary apoplexy. Preeclampsia, a serious condition unique to pregnancy, must also be considered. A high index of suspicion for carbon monoxide toxicity should be maintained. Primary headaches should be a diagnosis of exclusion. When advanced imaging is indicated, magnetic resonance imaging (MRI) should be used, if available, to reduce radiation exposure. Contrast agents should be avoided unless absolutely necessary. Medical therapy should be selected with careful consideration of adverse fetal effects. Herein, we present a review of the literature and discuss an approach to the evaluation and management of headache in pregnancy

## INTRODUCTION

A 30-year-old pregnant female at 10 weeks gestational age (GA) presented to the emergency department (ED) complaining of headache. The headache was sudden onset at maximal intensity and was described as left-sided pressure. There was associated lightheadedness, dizziness, blurred vision, and numbness and weakness of her right arm. There was no trauma or loss of consciousness, no slurred speech, and no other numbness or weakness. Approximately 90 minutes after headache onset, she reported spontaneous improvement in headache intensity from 10/10 to 6/10, resolution of the numbness and weakness of her right arm, and persistent blurred vision. She denied any prior similar episodes or history of migraine headache. She had no history of preeclampsia or other pregnancy-related complication. She had no associated fever, chills, or neck pain. A review of systems was otherwise negative. Past medical and surgical history was noncontributory. Medications included acetaminophen and prenatal vitamin. She smoked cigarettes, but denied alcohol or illicit drug use.

On examination she was awake, alert and oriented; Glasgow Coma Scale score was 15. She was afebrile with a blood pressure of 111/64mmHg and heart rate of 62 beats per minute. Cranial nerves were grossly intact, pupils were 3mm bilaterally and equally round and reactive to light, extraocular movements were intact, and no nystagmus was noted. She had a normal gait, 5/5 strength, and grossly intact sensation throughout the extremities. She had no cervical spinal tenderness and no meningismus. The remainder of her physical exam was unremarkable.

Laboratory studies revealed a leukocytosis of 13.6 × 10^9^ white blood cells/L and normal electrolytes. A non-contrast computed tomography (CT) of the head was negative. Subarachnoid hemorrhage (SAH) was a diagnosis of consideration in this case. Recent studies suggest that a negative head CT obtained within six hours of headache onset is sufficient to rule out SAH.^1^,^2^ Additionally, appropriate evaluation of cerebrospinal fluid (CSF) requires sufficient time for the development of xanthochromia.^3^,^4^ As the patient presented only 90 minutes after headache onset, a lumbar puncture (LP) was not immediately indicated for further evaluation of SAH. As cerebral venous thrombosis was also a diagnosis of consideration, a magnetic resonance image (MRI) with arteriography and venography (MRA and MRV) of the head was then obtained. Because the patient was pregnant, the study was obtained without gadolinium contrast. This showed no evidence of cerebral venous thrombosis, aneurysm, or vascular malformation, but did reveal a five-millimeter hemorrhagic appearing pituitary lesion consistent with pituitary apoplexy.

Headache is a common presenting complaint in the ED. The differential diagnosis is broad and includes benign and ominous etiologies. When evaluating the pregnant patient with headache, there are important unique considerations regarding the differential diagnosis, imaging options, and medical management. Herein, we present a review of the literature and discuss an approach to the evaluation and management of headache in pregnancy.

## ADVANCED IMAGING IN THE PREGNANT PATIENT

Advanced neuroimaging should be obtained for patients with sudden onset severe headache, rapidly increasing headache frequency or neurologic deficits, and should be considered for patients with new onset headache or headache that differs from baseline. Non-contrast head CT is the initial study of choice.

The estimated fetal radiation dose from a head CT is very low, <1 rad.^5^–^7^ At eight to fifteen weeks GA, the fetus is at greatest risk for radiation-related injury. A fetal dose of 1–2 rad may increase the risk of leukemia to 1 in 2,000 children compared to 1 in 3,000 children for the general population. The risk of fetal anomalies, growth restriction, or abortion is not increased with radiation doses of <5 rad.^5^,^6^ Most CT contrast agents contain iodine derivatives, which have not been formally studied in pregnancy. However, neonatal hypothyroidism has been associated with some iodinated agents taken in early pregnancy. Contrast should be avoided unless absolutely necessary.^5^–^8^

MRI involves no radiation and is safe in pregnancy.^5^ MRI is recommended for adequate imaging of the posterior fossa, and MRA and MRV are recommended to rule out vascular events.^7^ Gadolinium contrast crosses the placenta, and animal studies have demonstrated adverse fetal effects. Gadolinium should therefore be avoided unless absolutely necessary to confirm the diagnosis.^5^,^7^,^8^ Although contrast MRA/MRV provides superior imaging, non-contrast MRA/MRV produces quality images and is typically sufficient for establishing a diagnosis.^9^

## EVALUATION AND MANAGEMENT

### Etiologies for which pregnancy increases risk

#### Preeclampsia

Preeclampsia is defined as hypertension (systolic blood pressure >140mmHg OR diastolic blood pressure >90mmHg) *and* proteinuria (>0.3g protein in a 24-hour urine collection) at >20 weeks GA in a woman who was known to be normotensive prior to pregnancy. It is considered severe at blood pressures >160/>110mmHg. Preeclampsia occurs in approximately 2–8% of healthy nulliparous pregnancies. The risk is higher in African American-women, women over age 35 years, multifetal gestations, and women with a history of preeclampsia, hypertension, diabetes, and obesity, among others. Patients may present with headache, visual disturbances, edema, and epigastric pain. Severe preeclamptics may present with multi-organ involvement (e.g., pulmonary edema, oliguria, thrombocytopenia, elevated liver enzymes, persistent severe headache, blurred vision, blindness, or altered mental status). Preeclamptics with new onset grand mal seizure are considered to have eclampsia.^10^,^11^

The evaluation for preeclampsia should include an evaluation for HELLP (hemolysis, elevated liver enzymes, low platelets) syndrome with complete blood count and liver function tests. A urine dip with 1+ protein is suggestive of preeclampsia but not diagnostic; a 24-hour urine collection should be arranged.^10^,^11^

Obstetrics should be consulted for all patients with mild to moderate preeclampsia as these patients require maternal/fetal monitoring. Severe preeclamptics should be hospitalized and the fetus monitored. Intravenous magnesium should be given to prevent seizure. Typical dosing consists of a 4–6g load infused over 15–20 minutes followed by 2g/hr infusion. Patients who are not actively seizing may not require loading.^10^,^11^ Antihypertensives should also be given to maintain blood pressures of 140–155/90–105 mmHg. Recommended agents include hydralazine and labetalol. Hydralazine is given intravenously 5–10mg every 15–20 minutes as needed to achieve goal pressures. Labetalol is given intravenously in a 20mg bolus dose, followed by 40mg and then 80mg every 10 minutes to a maximum dose of 220mg.^10^ Delivery of the fetus should be considered if the gestational age is appropriate or if cerebral symptoms or severe uncontrolled blood pressures persist despite maximum antihypertensive and magnesium therapy, regardless of gestational age. Corticosteroids can be given to accelerate fetal lung maturity if <34 weeks GA.^11^

#### Cerebral venous thrombosis

The risk of cerebral venous thrombosis (CVT) is increased in the prothrombotic pregnant state. Most pregnancy-related CVT occurs in the third trimester.^12^,^13^ Common presenting complaints include headache, focal neurologic deficit, seizure, altered mental status, and signs of elevated intracranial pressure (ICP) such as papilledema. The headache is typically sub-acute, although thunderclap headache has been reported. Headache with focal neurologic findings or seizures should increase suspicion of CVT, particularly in the peripartum population.^12^–^15^

The diagnosis of CVT is based on neuroimaging. Classic non-contrast CT findings are the empty delta sign, dense triangle sign, or cord sign.^13^–^15^ More often, CT will show nonspecific edema or infarction, but the imaging may be normal in up to 30% of cases, particularly if obtained within the first 5–10 days of symptom onset. MRI/MRV is the imaging modality of choice.^13^–^15^ Non-contrast MRV is commonly used to evaluate for CVT; however, contrast CT venography or MRV may be required to detect acute thrombus (up to 5 days old).^9^

Anticoagulation is the standard of care for management of CVT. Low molecular weight heparin (LMWH) does not cross the placenta and is the first-line treatment for anticoagulation in pregnancy. Anticoagulation should be continued throughout the pregnancy and post-partum for a minimum of six weeks for a total minimum duration of therapy of three to six months. Although warfarin is contraindicated in pregnancy, post-partum patients may be safely transitioned to oral warfarin. Elevated ICP should be treated if present.^12^–^16^

#### Pituitary apoplexy

Pituitary apoplexy is caused by acute ischemic or hemorrhagic infarction as the pituitary gland expands and outgrows its blood supply or compresses the vessels against the sella.^12^,^17^ It is most commonly seen in men over the age of 50 years and in a pre-existing pituitary adenoma.^17^,^18^ Although rare, the massive hyperplasia of lactotrophs that occurs in pregnancy causes the pituitary gland to grow by as much as 130% putting pregnant patients at risk.^19^–^21^ Patients will present most commonly with sudden onset severe headache and nausea and vomiting and less commonly with visual symptoms, altered mental status, or coma.^17^–^19^,^22^ Secondary adrenal insufficiency can occur, causing severe hypotension and hyponatremia which can be life-threatening.^18^

In pituitary apoplexy, CT may demonstrate a recent bleed or hyperdense lesion in the pituitary. MRI is more sensitive for delineating the relationship between the pituitary and surrounding structures.^17^,^18^

Pituitary apoplexy patients with persistent visual symptoms, neurologic deficit, or altered mental status require urgent surgical decompression.^17^,^18^,^22^ Secondary adrenal insufficiency should be treated immediately with fluid and electrolyte replacement and hydrocortisone.^17^–^19^

#### Subarachnoid hemorrhage

Subarachnoid hemorrhage occurs most commonly as the result of a ruptured aneurysm or arterial-venous malformation (AVM). In pregnancy, AVM rupture typically occurs early (15–20 weeks GA) and in younger women (20–25 years), while aneurysm rupture usually occurs later (30–40 weeks GA) and in older women (30–35 years). A theoretical increased risk in pregnancy has been proposed. The cardiac output and blood volume peak of the third trimester is thought to increase the risk of aneurysm rupture.^23^ However, other studies suggest there is no increased risk of SAH in pregnancy, labor, or delivery.^24^–^26^ Patients will classically present with a thunderclap headache, but nausea and vomiting, stiff neck, photophobia, syncope, and focal neurologic deficit may also be seen. Patients with a preceding less severe headache episode should raise concern for a sentinel bleed.^26^

Evaluation of SAH should begin with a non-contrast head CT. If negative, a LP should be performed and the CSF examined for xanthrochromia. If necessary, CT angiogram (CTA) may localize the lesion (e.g. aneurysm, AVM) or document vasospasm.^26^

Patients with SAH should be hospitalized to facilitate early neurosurgical intervention with clipping or coiling to reduce the risk of rebleeding.^23^,^26^

#### Arterial dissection

Vertebral artery dissection (VAD) and carotid artery dissection (CAD) are common causes of cerebral vascular accident in young patients. Patients often present with unilateral headache or neck pain followed by signs of posterior circulation ischemia (e.g., Horner’s syndrome, diplopia, delayed transient ischemic attack, and stroke).^27^–^29^ In the general population, the incidence of spontaneous CAD is estimated at 2.5–3/100,000 and spontaneous VAD is estimated at 1–1.5/100,000.^29^–^31^ Pregnancy theoretically increases the risk for spontaneous dissection. In pregnancy, high progesterone and/or decreased collagen synthesis is thought to weaken the arterial wall. This, together with increased sheer stress on the vessel wall caused by increased intravascular volume and cardiac output, is thought to increase the risk of dissection in the pregnant population.^27^,^32^ However, the reported incidence of VAD (1.5/100,000) in the pregnant population is comparable to that of the general population; less is known about the incidence of CAD in pregnancy.^27^,^33^

Intra-arterial angiography is the gold standard for diagnosis of arterial dissection, typically revealing an intimal flap or double lumen, aneurysm, occlusion, or the classic “string sign.”^29^ Non-contrast MRA is the preferred modality in the pregnant patient, however.^27^

As in CVT, anticoagulation is the standard of care for management of arterial dissection. LMWH is again the first-line treatment and the duration of anticoagulation is the same as for CVT. Post-partum patients may be transitioned to oral warfarin. Elevated ICP should be treated if present.^12^–^16^ Anticoagulation is contraindicated in patients with intracranial extension of the dissection, intracranial aneurysm, or infarction with hemorrhagic transformation or mass effect. Patients without ischemic symptoms may be treated with an antiplatelet agent only.^34^ Aspirin (pregnancy category D) has been demonstrated safe and effective at low doses (< 100mg/day).^16^ Patients with evidence of hemodynamic insufficiency due to severe stenosis or occlusion may require intervention to increase cerebral blood flow (e.g.; induced hypertension, volume expansion, or emergent endovascular repair or stenting).^29^ Endovascular repair or stenting in stable patients should be reserved for those in whom anticoagulation is contraindicated.^34^

### Other Secondary Headaches

#### Idiopathic Intracranial Hypertension

Idiopathic intracranial hypertension (IIH), formerly pseudotumor cerebri, is defined as an opening pressure of >250 mmH_2_O with normal CSF. IIH classically affects obese women of childbearing age and is relatively rare, occurring in approximately 0.9 per 100,000 in the general population and 4–19 per 100,000 in obese women.^35^ Occurrence rates are estimated at 5% in the pregnant population.^20^ Outcomes of pregnant women with IIH are the same as those of non-pregnant women, and pregnancy outcome is unaffected.^35^ Patients commonly present with headache (90%) and visual changes (e.g., transient visual obscurations, diplopia, and blindness). Papilledema is almost universally present and can occasionally be unilateral.^35^–^37^ In patients without papilledema, a history of headache with visual disturbances, tinnitus, or sixth-nerve palsies is very suggestive of IIH.^37^

Evaluation for IIH begins with imaging to rule out other causes of intracranial hypertension with non-contrast head CT or MRI. It is important to consider that CVT also presents with headache and papilledema; a diagnosis of CVT should be excluded in these patients. A thorough review of the patient’s medications may reveal one of a number of agents known to cause intracranial hypertension including vitamin A and nitrofurantoin.^35^

First-line therapy for IIH in pregnancy is diet and weight control.^35^ Second-line therapies include serial LPs and/or acetazolamide (category C). Acetazolamide use in pregnancy has been limited because of teratogenic potential. However, Falardeau et al 2013^38^ reviewed outcomes of pregnant women with IIH treated with acetazolamide and found no forelimb or axial skeletal abnormalities and no difference in the abortion rate, minor complication rate, or minor abnormality rate compared to a control group of pregnant women with IIH who did not use acetazolamide. These data suggest acetazolamide can be safely used in pregnancy; dosing starts at 0.5–1g/day in divided doses to a maximum of 2g/day. The use of other diuretics is controversial because of the potential for decreased placental blood flow.^35^ Steroids (category C) are reserved for urgent short-term treatment in patients awaiting surgery. Surgical therapy is recommended for those with severe or progressive visual loss despite medical management. Optic nerve sheath fenestration creates a window in the optic nerve sheath allowing CSF to drain into the retrobulbar space, directly protecting the optic nerve. Lumboperitoneal or ventriculoperitoneal shunting is also an option however over 50% become occluded, infected, or migrate requiring reoperation.^35^ Visual outcomes for pregnant patients with IIH are the same as those in the nonpregnant IIH population.^35^,^36^

#### Meningitis

Meningitis in pregnancy presents similarly to that in the non-pregnant population with headache, fever, nausea, vomiting, nuchal rigidity, and/or altered mental status.^39^–^41^ Otitis and sinusitis infection often precede meningitis in pregnancy.^40^,^42^ *Streptococcus pneumoniae* and *Listeria monoctogenes* are the most common causative organisms and are associated with a very high mortality rate (28%). Miscarriages and neonatal death are also common consequences of meningitis in pregnancy.^42^

If meningitis is suspected, blood cultures and a LP should be obtained. A CT of the head should be done prior to the LP if there are any concerns for elevated ICP. Studies suggest that once antimicrobial therapy is started, sterilization of the CSF occurs within two hours for *N. meningitides* infections, and within four hours for *S. pneumoniae* infections; however, antibiotics should not be delayed if LP cannot be performed early.^39^

Empiric antimicrobial therapy for meningitis consists of a third-generation cephalosporin, such as cefotaxime or ceftriaxone (category B), and vancomycin (category C).^39^,^40^,^42^ Because *Listeria* infection is common in pregnancy, ampicillin (category B) should also be given.^42^ If viral causes are suspected, add acyclovir (category B). Studies suggest that prognosis in viral meningitis is directly related to the delay in empiric acyclovir administration.^43^ Some data suggest adjunct therapy with steroids reduces mortality. Dexamethasone has few side effects in third trimester pregnancy (category C) and may also be used.^39^,^42^

#### Carbon Monoxide Toxicity

Carbon monoxide (CO) toxicity presents with non-specific signs and symptoms, but headache is present in the vast majority (approximately 84%). Other symptoms include weakness, nausea, confusion, and shortness of breath, among others.^44^ In addition to maternal toxicity, fetal toxic effects include teratogenicity, neurological dysfunction, decreased birth weight, increased fetal death, and premature closure of the *ductus arteriosus*.^45^ The fetal carboxyhemoglobin (COHbg) level cannot be accurately estimated from the maternal COHgb level. The fetal level is higher than the maternal level at baseline and rises faster and clears more slowly than the maternal level.^45^,^46^ The fetal outcome is proportionate to the severity of maternal toxicity; the risk is very high when the mother shows signs of altered mental status.^46^

Because the presentation of CO toxicity is non-specific, a high index of suspicion should be maintained. Normal COHgb levels in a nonsmoker are <2% and in smokers are 5–13%. Pulse oxyimetry cannot distinguish between oxyhemoglobin and carboxyhemoglobin and so is not a reliable measure of CO toxicity.^44^

All patients with suspected CO toxicity should receive 100% high flow oxygen. This reduces the half-life of CO from five hours to one hour. Some recommend hyperbaric oxygen (HBO) therapy for pregnant women with any signs of acute poisoning or COHgb level >20%.^46^ Others recommend HBO therapy for COHgb >20%, or neurologic effects or signs of fetal distress regardless of COHgb level.^45^ Because the fetal half-life of COHgb is longer than the maternal half-life, the duration of HBO therapy in pregnant women should be longer than in non-pregnant women.^46^ If HBO therapy is not available, give 100% high flow oxygen for five times as long as is needed to reduce the maternal COHgb level to normal. Repeat treatments should be considered if neurological symptoms or fetal distress persists 12 hours after the first treatment.^45^

### Primary Headaches

#### Migraine Headache

Migraine headache (MH) typically presents with unilateral, pulsating headache that is aggravated by physical activity and associated with nausea and vomiting and/or photophobia or phonophobia. Some patients experience a preceding aura consisting of a reversible focal neurologic deficit.^47^ Typically it is not the MH symptom complex, but the frequency and severity of attacks that changes in pregnancy. In general, 50 to 75% of women have a reduction in frequency of attacks during pregnancy. Migraine headache is known to be influenced by cyclical changes in reproductive hormones, and it is thought that the absence of hormonal fluctuation during pregnancy is responsible for the observed improvement. In contrast, approximately 8% of women experience increased frequency or intensity of MH during pregnancy.^48^–^50^ None of these studies demonstrate a difference in incidence or course of MH between primigravid and multiparous women. New-onset MH can also occur in up to 16.5% of pregnant women and usually presents in the first trimester.^49^

Treatment of acute migraine headache is challenging. First-line therapy typically includes a non-narcotic analgesic and an antiemetic. Several of the common initial agents (acetaminophen/aspirin/caffeine, ketorlac, prochlorperazine) are contraindicated in pregnancy.^7^,^51^,^52^ Prochlorperazine is associated with congenital heart defects and cleft palate and is contraindicated.^7^ A recent study also demonstrates an increased risk of cleft palate with the use of ondansetron (category B).^53^ Although studies investigating droperidol are few, there are no significant differences in major or minor birth defect rates compared to the general population.^54^,^55^ Dihydroergotamine (DHE), valproic acid infusion and sumatriptan, are common second-line agents for migraine. In pregnancy, DHE is contraindicated because its vasoconstrictive effects decrease uterine blood flow.^7^,^52^ Valproic acid is known to cause spina bifida and other fetal anomalies and is also contraindicated in pregnancy.^7^,^55^,^56^ Some studies suggest that sumatriptan is associated with low birth weight, preterm delivery, and minor fetal anomalies while others suggest that there is no increased risk compared to the general population.^57^–^59^ Propofol is an emerging treatment option for migraine and is pregnancy category B.^60^ Dexamethasone has been used to prevent migraine recurrence, but is associated with increased risk of cleft lip and palate in first trimester exposures, so should not be used early in pregnancy.^53^,^54^ Treatment options for migraine headache are summarized in Table 2. A combination of intravenous fluids and droperidol as initial therapy, followed by propofol or sumatriptan as second line agents, is a reasonable approach.

#### Cluster Headache

Cluster headache (CH) is described as a severe and unilateral, orbital, supraorbital, and/or temporal headache; attacks occur up to eight times daily and are associated with ipsilateral autonomic dysfunction (e.g., conjunctival injection, lacrimation, rhinorrhea, miosis, etc.).^47^ Data regarding CH in pregnancy are limited and conflicting.^63^–^65^

First-line treatment for acute cluster headache (CH) attack in pregnancy remains high flow oxygen.^63^,^64^ About 60% of all CH patients have significant reduction in pain after 30 minutes of oxygen therapy. Sumatriptan, effective in 2/3 of CH patients, is an option, although its safety profile in pregnancy is not clear, as previously discussed. Ipsilateral intranasal lidocaine (pregnancy category B) has been used with rapid relief and no significant general side effects and is effective in 1/3 of CH cases.^61^ Again, DHE is contraindicated.^61^,^63^

#### Tension Type Headache

Tension type headache (TTH) is the most common and least well-studied primary headache. TTH is typically bilateral and of pressing or tightening quality. It is not exacerbated by routine physical activity and is not associated with nausea, but may be associated with photophobia or phonophobia.^47^ Data about the incidence of TTH in pregnancy is limited, but some authors suggest that the majority of TTH sufferers are not affected by pregnancy.^48^

Tension type headache can be treated with over-the-counter (OTC) analgesics. Acetaminophen is safe during pregnancy and is the OTC analgesic of choice. Salicylates and non-steroidal anti-inflammatory agents are associated with adverse fetal effects and should be avoided.^7^,^51^

## SUMMARY

The objective of the ED evaluation of headache is to rule out ominous secondary causes. The diagnosis and management of headache in the pregnant patient presents several challenges. Physiologic changes induced by pregnancy increase the risk of CVT, dissection, and pituitary apoplexy. Preeclampsia must also be considered. A high index of suspicion for CO toxicity should be maintained. Primary headaches should be a diagnosis of exclusion. When advanced imaging is indicated, MRI should be used whenever possible to reduce radiation exposure. If CT is necessary, imaging of the head rarely exceeds fetal radiation danger thresholds. Contrast agents do cause adverse fetal effects and should be avoided unless absolutely necessary for accurate diagnosis. An approach to the evaluation of the pregnant patient with headache is proposed (Figure). Medical therapy should be selected with careful consideration of adverse fetal effects. The management of secondary headache is tailored to the etiology (Table 1) as are treatment options for primary headache (Table 2).

## Figures and Tables

**Figure:**
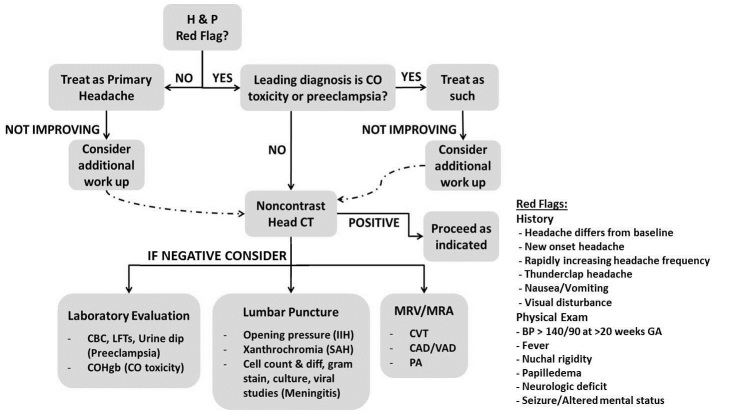
Suggested approach to evaluation of headache in a pregnant patient. Leading differential diagnosis and/or availability of advanced imaging should determine the order of laboratory evaluation, lumbar puncture, and/or MRV/MRA. Additional laboratory studies may be obtained if indicated based on differential diagnosis. *H&P*, history and physical; *COHgb*, carboxyhemoglobin; *CO*, carbon monoxide; *CT,* computed tomography; *CBC,* complete blood count; *LFT,* liver function test; *IIH*, idiopathic intracranial hypertension; *diff*, differential; *SAH*, subarachnoid hemorrhage; *MRV,* magnetic resonance venogram; *MRA*, magnetic resonance angiogram; *CVT*, cerebral venous thrombosis; *CAD,* carotid artery dissection; *VAD,* vertebral artery dissection; *PA*, pituitary apoplexy; *BP,* blood pressure; *GA,* gestational age

**Table 1:** Medications for treatment of secondary headache in pregnancy.

Disease	Treatment	Medication	Category* (1^st^/2^nd^/3^rd^)	Adverse effect(s)	Recommendation
Preeclampsia^10^–^11^,^66^	Antiepileptic	Magnesium	D/D/D	Neonatal respiratory or neuromuscular depression (transient)	Use with caution, Acceptable in some circumstances (i.e. preeclampsia)
	Antihypertensive	Hydralazine	C/C/C	Associated with increased rates of placental abruption and cesarean section, maternal headache, palpitations, hypotension, tachycardia	Use with caution
		Labetalol	C/C/C	Increased incidence of intrauterine growth restriction, rare cases of neonatal hypoglycemia, bradycardia, and hypotension	Use with caution
	Fetal lung maturity	Corticosteroids	C/C/C	Cleft lip and palate (first trimester use)	Not recommended in 1^st^ trimester pregnancy
Cerebral venous thrombosis and arterial dissection^12^–^16^,^34^,^66^	Anticoagulation	Low molecular weight Heparin (LMWH)	B/B/B	Maternal heparin induced thrombocytopenia and/or osteoporosis; no documented increased fetal risk	Acceptable
		Unfractionated heparin	C/C/C	Maternal heparin induced thrombocytopenia and/or osteoporosis; no documented increased fetal risk	Acceptable
		Warfarin	X/X/X	“Warfarin embryopathy,” intracranial hemorrhage, intrauterine fetal demise, pregnancy loss, maternal hemorrhage	Contraindicated
	Antiplatelet	Clopidogrel	B/B/B	Maternal bleeding; no documented increased fetal risk	Use with caution
		Aspirin	C/C/D	Premature closure of *ductus arteriosus*, oligohydramnios (especially 3^rd^ trimester use)	Use with caution; not recommended in 3^rd^ trimester
Pituitary Apoplexy^17^–^19^,^66^	Secondary adrenal insufficiency therapy	Fluid/Electrolyte replacement	--/--/--	No documented increased fetal risk	Acceptable
		Hydrocortisone	C/C/C	Cleft lip and palate (first trimester use)	Not recommended in 1^st^ trimester
Subarachnoid Hemorrhage 51,66	Analgesia	Acetaminophen	C/C/C	No documented increased fetal risk	Acceptable
		Opiates	C/C/C	Maternal respiratory depression, histamine release, nausea	Acceptable
Idiopathic Intracranial Hypertension 35,38,66	Reduce intracranial pressure	Acetazolamide	C/C/C	No documented increased fetal risk	Acceptable
		Topiramate	D/D/D	Cleft lip and palate	Not recommended
		Furosemide	C/C/C	Maternal hypovolemia and decreased placental perfusion	Not recommended
		Thiazides	B/B/B	Maternal and neonatal hyponatremia, hypokalemia, hyperglycemia, thrombocytopenia; smooth muscle contraction and initiation of labor	Not recommended
		Steroids	C/C/C	Cleft lip and palate (first trimester use)	Not recommended in 1^st^ trimester
Meningitis^39^–^40^,^42^,^66^	Antibiotic	3^rd^ generation cephalosporin	B/B/B	No documented increased fetal risk	Acceptable
		Vancomycin	C/C/C	Potential neonatal ototoxicity and nephrotoxicity, No documented increased fetal risk	Acceptable
		Ampicillin	B/B/B	No documented increased fetal risk	Acceptable
	Antiviral	Acyclovir	B/B/B	No documented increased fetal risk	Acceptable
	Other	Dexamethasone	C/C/C	Cleft lip and palate (first trimester use)	Not recommended in 1^st^ trimester
Carbon MonoxideToxicity^45^–^46^,^66^	Oxygen therapy	100% High flow oxygen	--/--/--	No documented increased fetal risk	Acceptable
		Hyperbaric Oxygen therapy (HBO)	--/--/--	No documented increased fetal risk	Acceptable

* Adapted from www.drugs.com/pregnancy.^46^

**Table 2:** Medications for treatment of primary headache in pregnancy.

Disease	Treatment	Medication	Category* (1^st^/2^nd^/3^rd^)	Adverse effect(s)	Recommendation
Migraine Headache 7,52–56,60,66	Analgesia	Ketorolac	C/C/D	Premature closure of *ductus arteriosus*, oligohydramnios (especially in 3^rd^ trimester use)	Use with caution; not recommended in 3^rd^ trimester
		Acetaminophen/Aspirin/Caffeine	D/D/D	Neonatal hemorrhage, decreased birth weight, birth defects	Not Recommended
	Antiemetic	Metoclopramide	B/B/B	No documented increased fetal risk	Acceptable
		Ondansetron	B/B/B	Possible increased risk of cleft palate, risk of maternal prolonged QTc and arrhythmia	Use with caution
		Promethazine	C/C/C	No documented increased fetal risk	Acceptable
		Droperidol	C/C/C	No documented increased fetal risk, risk of maternal prolonged QTc and arrhythmia	Use with caution
		Prochlorperazine	C/C/C	Congenital heart defects, cleft palate	Not Recommended
	Other	Propofol	B/B/B	Neonatal hypotonia and sedation (us ually transient), maternal hypotension and respiratory depression	Acceptable
		Sumatriptan	C/C/C	Low birth weight, preterm delivery, minor fetal anomalies	Use with caution
		Dexamethasone	C/C/C	Cleft lip and palate (first trimester use)	Not recommended in 1^st^ trimester
		Dihydroergotamine	X/X/X	Vasoconstriction, decreased uterine blood flow	Contraindicated
		Valproic acid	X/X/X	Spina bifida and other fetal anomalies	Contraindicated
Cluster Headache 61,63–64,66	Other	High Flow Oxygen	--/--/--	No documented increased fetal risk	Acceptable
		Intranasal lidocaine	B/B/B	No documented increased fetal risk	Acceptable
		Sumatriptan	C/C/C	Low birth weight, preterm delivery, minor fetal anomalies	Use with caution
		Dihydroergotamine	X/X/X	Vasoconstriction, decreased uterine blood flow	Contraindicated
Tension Type Headache 51,66	Analgesia	Acetaminophen	C/C/C	No documented increased fetal risk	Acceptable
		NSAIDs	C/C/D	Premature closure of *ductus arteriosus*, oligohydramnios (especially 3^rd^ trimester use)	Use with caution; not recommended in 3^rd^ trimester
		Salicylates	D/D/D	Neonatal hemorrhage, decreased birth weight, birth defects	Not Recommended

* Adapted from www.drugs.com/pregnancy.^46^

**Table 3:** U.S. Food and Drug Administration pregnancy categories.

Pregnancy category	Description
A	Adequate and well-controlled studies in pregnant women have failed to demonstrate a risk to the fetus in the first trimester of pregnancy (and there is no evidence of a risk in later trimesters)
B	Animal reproduction studies have failed to demonstrate a risk to the fetus and there are no adequate and well-controlled studies in pregnant women
C	Animal reproduction studies have shown an adverse effect on the fetus and there are no adequate and well-controlled studies in humans, but potential benefits may warrant use of the drug in pregnant women, despite potential risks
D	There is positive evidence of human fetal risk based on adverse reaction data from investigational or marketing experience or studies in humans, but potential benefits may warrant use of the drug in pregnant women despite potential risks
X	Studies in animals or humans have demonstrated fetal abnormalities and/or there is positive evidence of human fetal risk based on adverse reaction data from investigational or marketing experience, and the risks involved in use of the drug in pregnant women clearly outweigh potential benefits

Adapted from www.drugs.com/pregnancy.^46^

## References

[1] Perry JJ, Stiell IG, Sivilotti MLA (2011). Sensitivity of computed tomography performed within six hours of onset of headache for diagnosis of subarachnoid hemorrhage: prospective cohort study. BMJ.

[2] Backes D, Rinkel GJE, Kemperman H (2012). Time-dependent test characteristics of head computed tomography in patients suspected of nontraumatic subarachnoid hemorrhage. Stroke.

[3] Edlow JA, Caplan LR (2000). Avoiding pitfalls in the diagnosis of subarachnoid hemorrhage. N Engl J Med.

[4] Edlow JA, Bruner KS, Horowitz GL (2002). Xanthochromia: A survey of laboratory methodology and its clinical implications. Arch Pathol Lab Med.

[5] ACOG Committee Opinion #299. American College of Obstetricians and Gynecologists (2004). Guidelines for diagnostic imaging during pregnancy. Obstet Gynecol.

[6] Miller JC, Lee SI (2004). Risks from ionizing radiation in pregnancy. Radiology Rounds.

[7] Contag SA, Mertz HL, Bushnell CD (2009). Migraine during pregnancy: is it more than a headache?. Nat Rev Neurol.

[8] Puri A, Khadem P, Ahmed S (2012). Imaging of trauma in a pregnant patient. Semin Ultrasound CT MRI.

[9] Leach JL, Fortuna RB, Jones BV (2006). Imaging of cerebral venous thrombosis: current techniques, spectrum of findings, and diagnostic pitfalls. Radiographics.

[10] American College of Obstetrics and Gynecologists (2002). Diagnosis and management of preeclampsia and eclampsia. ACOG Practice Bulletin No. 33. Obstet Gynecol.

[11] Sibai BM (2003). Diagnosis and management of gestational hypertension and preeclampsia. The Obstet Gynecol.

[12] Bousser MG, Crassard I (2012). Cerebral venous thrombosis, pregnancy and oral contraceptives. Thromb Res.

[13] Demir CF, Inci MF, Ozkan F (2013). Clinical and radiological management and outcome of pregnancies complicated by cerebral venous thrombosis: a review of 19 cases. J Stroke Cerebrovasc Dis.

[14] Bousser MG (2000). Cerebral venous thrombosis: diagnosis and management. J Neurol.

[15] Masuhr F, Mehraein S, Einhaupl K (2004). Cerebral venous and sinus thrombosis. J Neurol.

[16] Gibson PS, Powrie R (2009). Anticoagulants and pregnancy: when are they safe?. Cleve Clin J Med.

[17] Ranabir S, Baruahl MP (2011). Pituitary apoplexy. Indian J Endocrinol Metab.

[18] Chanson P, Lepeintre JF, Ducreux D (2004). Management of pituitary apoplexy. Expert Opin Pharmacother.

[19] de Heide LJM, van Tol KM, Doorenbos B (2004). Pituitary apoplexy presenting in pregnancy. Neth J Med.

[20] Ginath S, Golan A (2010). Gestational pituitary–tumor apoplexy. N Engl J Med.

[21] Karaca Z, Tanriverdi F, Unluhizarci K (2010). Pregnancy and pituitary disorders. Eur J Endocrinol.

[22] Ayuk J, McGregor EJ, Mitchell RD (2004). Acute management of pituitary apoplexy – surgery or conservative management?. Clin Endocrinol (Oxf).

[23] Singer JR, Hummelgard AB, Martin EM (1985). Ruptured aneurysm in pregnancy. J Neurosurg Nurs.

[24] Algra AM, Klijn CJM, Helmerhorst FM (2012). Female risk factors for subarachnoid hemorrhage: a systematic review. Neurology.

[25] Bateman BT, Olbrecht VA, Berman MF (2012). Peripartum subarachnoid hemorrhage: nationwide data and institutional experience. Anesthesiology.

[26] Connolly ES, Rabinstein AA, Carhuapoma JR (2012). Guidelines for the management of aneurysmal subarachnoid hemorrhage: a guideline for healthcare professionals from the American Heart Association/American Stroke Association. Stroke.

[27] Cenkowski M, daSilvia M, Bordun KA (2012). Spontaneous dissection of the coronary and vertebral arteries post-partum: case report and review of the literature. BMC Pregnancy and Childbirth.

[28] Haneline MT, Lewkovich GN (2005). An analysis of the etiology of cervical artery dissections: 1994 to 2003. J Manipulative Physiol Ther.

[29] Redekop GJ (2008). Extracranial carotid and vertebral artery dissection: a review. Can J Neurol Sci.

[30] Dziewas R, Konrad C, Drager B (2003). Cervical artery dissection – clinical features, risk factors, therapy an outcome in 126 patients. J Neurol.

[31] Lee VH, Brown RD, Mandrekar JN (2006). Incidence and outcome of cervical artery dissection: a population-based study. Neurology.

[32] Tuluc M, Brown D, Goldman B (2006). Lethal vertebral artery dissection in pregnancy: a case report and review of the literature. Arch Pathol Lab Med.

[33] Maderia LM, Hoffman MK, Schlossman PA (2007). Internal carotid artery dissection as a cause of headache in the second trimester. Am J Obstet Gynecol.

[34] Schievnick WI (2000). The treatment of spontaneous carotid and vertebral artery dissections. Curr Opin Cardiol.

[35] Kesler A, Kupferminc M (2013). Idiopathic intracranial hypertension and pregnancy. Obstet Gynecol.

[36] Huna-Baron R, Kupersmith MJ (2002). Idiopathic intracranial hypertension in pregnancy. J Neurol.

[37] Jones JS, Nevai J, Freeman MP (1999). Emergency department presentation of idiopathic intracranial hypertension. Am J Emerg Med.

[38] Falardeau J, Lobb BM, Golden S (2013). The use of acetazolamide during pregnancy in intracranial hypertension patients. J Neuroophthalmol.

[39] Bhimraj A (2012). Acute community-acquired bacterial meningitis in adults: an evidenice-based review. Cleve Clin J Med.

[40] Landrum LM, Hawkins A, Goodman JR (2007). Pneumococcal meningitis during pregnancy: a case report and review of literature. Infect Dis Obstet Gynecol.

[41] Schaap TP, Schutte JM, Zwart JJ (2012). Fatal meningitis during pregnancy in the Netherlands: a nationwide confidential enquiry. BJOG.

[42] Adriani KS, Brouwer MC, van der Ende A (2012). Bacterial meningitis in pregnancy: report of six cases and review of the literature. Clin Microbiol Infec.

[43] Raschilas F, Wolff M, Delatour F (2002). Outcome of prognostic factors for herpes simplex encephalitis in adult patients: results of a multicenter study. Clin Infect Dis.

[44] Prockop LD, Chichkova RI (2007). Carbon monoxide intoxication: an updated review. J Neurol Sci.

[45] Van Hoesen KB, Camporesi EM, Hage ML (1989). Should hyperbaric oxygen be used to treat the pregnant patient for acute carbon monoxide poisoning? A case report and literature review. JAMA.

[46] Aubard Y, Magne I (2000). Carbon monoxide poisoning in pregnancy. BJOG.

[47] Headache Classification Subcommittee of the International Headache Society (2004). The international classification of headache disorders, 2^nd^ edition. Cephalagia.

[48] Karli N, Baykan B, Ertas M (2012). Impact of sex hormonal changes on tension-type headache and migraine: a gross-sectional population-based survery in 2,600 women. J Headache Pain.

[49] Kvisvik EV, Stovner LJ, Helde G (2011). Headache and migraine during pregnancy and puerperium: the MIGRA-study. J Headache Pain.

[50] Sances G, Granella F, Nappi RE (2003). Course of migraine during pregnancy and postpartum: a prospective study. Cephalagia.

[51] Black RA, Hill DA (2003). Over-the-counter medications in pregnancy. Am Fam Physician.

[52] Aukerman G, Kuntson D, Miser WF (2002). Management of the acute migraine headache. Am Fam Physician.

[53] Anderka M, Mitchell AA, Louik C (2012). Medications used to treat nausea and vomiting of pregnancy and the risk of selected birth defects. Birth Defects Res A Clin Mol Teratol.

[54] Nageotte MP, Briggs GG, Towers CV (1996). Droperidol and Diphendydramine in the management of hyperemesis gravidarum. Am J Obstet Gynecol.

[55] Ornoy A (2009). Valproic acid in pregnancy: how much are we endangering the embryo and fetus?. Reprod Toxicol.

[56] Mathew NT, Kailasam J, Meadors L (2000). Intravenous Valproate Sodium (Depacon) aborts migraine rapidly: a preliminary report. Headache.

[57] Soldin OP, Dahlin J, O’Mara DM (2008). Triptans in pregnancy. Ther Drug Monit.

[58] Cunnington M, Ephross S, Curchill P (2009). The safety of sumatriptan and naratriptan in pregnancy: what have we learned?. Headache.

[59] Nezvalova-Henriksen K, Spigset O, Nordeng H (2010). Triptan exposure during pregnancy and the risk of major congenital malformations and adverse pregnancy outcomes: results from the Norwegian mother and child cohort study. Headache.

[60] Soleimanpour H, Ghafouri RR, Taheraghdam A (2012). Effectiveness of intravenous Dexamethasone verses Propofol for pain relief in the migraine headache: a prospective double blind randomized clinical trial. BMC Neruology.

[61] Singh A, Alter H, Zaia B (2008). Does the addition of dexamethasone to standard therapy for acute migraine headache decrease the incidence of recurrent headache for patients treated in the Emergency Department? A Meta-analysis and systematic review of the literature. Acad Emerg Med.

[62] Carmichael SL, Shaw GM (1999). Maternal corticosteroid use and risk of selected congenital anomalies. Am J Med Genet.

[63] Calhun AH, Peterlin BL (2010). Treatment of cluster headache in pregnancy and lactation. Curr Pain Headache Rep.

[64] Giraud P, Chauvet S (2009). Cluster headache during pregnancy: case report and literature review. Headache.

[65] MacGregor EA (2012). Headache in pregnancy. Neurol Clin.

[66] www.Drugs.com/pregnancy.

